# Supplementary material for: Preparing for the interviewing process during Coronavirus disease-19 pandemic: Virtual interviewing experiences of applicants and interviewers, a systematic review
Source: PLoS One. 2020 Dec 7;15(12):e0243415. doi: 10.1371/journal.pone.0243415 (PMC7721161; doi:10.1371/journal.pone.0243415)
Supplement: S1 Appendix — (DOCX) [file pone.0243415.s001.docx]

**S1 Appendix. MeSH Used For PubMed Database.**

MeSH used for PubMed database is given below. Same strategy was used to perform search in other databases.

Limits: No date limit set. The database search was performed on May 31st, 2020.

**VIRTUAL RESIDENCY INTERVIEWS**

Search: **VIRTUAL RESIDENCY INTERVIEWS** Sort by: **Most Recent**

((((("virtual"[All Fields] OR "virtuality"[All Fields]) OR "virtualization"[All Fields]) OR "virtualized"[All Fields]) OR "virtualizing"[All Fields]) OR "virtuals"[All Fields]) AND ((((((((((((((("internship and residency"[MeSH Terms] OR ("internship"[All Fields] AND "residency"[All Fields])) OR "internship and residency"[All Fields]) OR "residencies"[All Fields]) OR "residency"[All Fields]) OR "reside"[All Fields]) OR "resided"[All Fields]) OR "residence"[All Fields]) OR "residence s"[All Fields]) OR "residences"[All Fields]) OR "residency s"[All Fields]) OR "resident"[All Fields]) OR "resident s"[All Fields]) OR "residents"[All Fields]) OR "resides"[All Fields]) OR "residing"[All Fields]) AND (("interview"[Publication Type] OR "interviews as topic"[MeSH Terms]) OR "interviews"[All Fields])

**Translations**

**VIRTUAL:** "virtual"[All Fields] OR "virtuality"[All Fields] OR "virtualization"[All Fields] OR "virtualized"[All Fields] OR "virtualizing"[All Fields] OR "virtuals"[All Fields]

**RESIDENCY:** "internship and residency"[MeSH Terms] OR ("internship"[All Fields] AND "residency"[All Fields]) OR "internship and residency"[All Fields] OR "residencies"[All Fields] OR "residency"[All Fields] OR "reside"[All Fields] OR "resided"[All Fields] OR "residence"[All Fields] OR "residence's"[All Fields] OR "residences"[All Fields] OR "residency's"[All Fields] OR "resident"[All Fields] OR "resident's"[All Fields] OR "residents"[All Fields] OR "resides"[All Fields] OR "residing"[All Fields]

**INTERVIEWS:** "interview"[Publication Type] .or. "interviews as topic"[MeSH Terms] .or. "interviews"[All Fields]

**VIDEOCONFERENCING RESIDENCY INTERVIEWS**

Search: **VIDEOCONFERENCING RESIDENCY INTERVIEWS** Sort by: **Most Recent**

(("videoconferenced"[All Fields] OR "videoconferencing"[MeSH Terms]) OR "videoconferencing"[All Fields]) AND ((((((((((((((("internship and residency"[MeSH Terms] OR ("internship"[All Fields] AND "residency"[All Fields])) OR "internship and residency"[All Fields]) OR "residencies"[All Fields]) OR "residency"[All Fields]) OR "reside"[All Fields]) OR "resided"[All Fields]) OR "residence"[All Fields]) OR "residence s"[All Fields]) OR "residences"[All Fields]) OR "residency s"[All Fields]) OR "resident"[All Fields]) OR "resident s"[All Fields]) OR "residents"[All Fields]) OR "resides"[All Fields]) OR "residing"[All Fields]) AND (("interview"[Publication Type] OR "interviews as topic"[MeSH Terms]) OR "interviews"[All Fields])

**Translations**

**VIDEOCONFERENCING:** "videoconferenced"[All Fields] OR "videoconferencing"[MeSH Terms] OR "videoconferencing"[All Fields]

**RESIDENCY:** "internship and residency"[MeSH Terms] OR ("internship"[All Fields] AND "residency"[All Fields]) OR "internship and residency"[All Fields] OR "residencies"[All Fields] OR "residency"[All Fields] OR "reside"[All Fields] OR "resided"[All Fields] OR "residence"[All Fields] OR "residence's"[All Fields] OR "residences"[All Fields] OR "residency's"[All Fields] OR "resident"[All Fields] OR "resident's"[All Fields] OR "residents"[All Fields] OR "resides"[All Fields] OR "residing"[All Fields]

**INTERVIEWS:** "interview"[Publication Type] .or. "interviews as topic"[MeSH Terms] .or. "interviews"[All Fields]

**ONLINE RESIDENCY INTERVIEWS**

Search: **ONLINE RESIDENCY INTERVIEWS** Sort by: **Most Recent**

"ONLINE"[All Fields] AND ((((((((((((((("internship and residency"[MeSH Terms] OR ("internship"[All Fields] AND "residency"[All Fields])) OR "internship and residency"[All Fields]) OR "residencies"[All Fields]) OR "residency"[All Fields]) OR "reside"[All Fields]) OR "resided"[All Fields]) OR "residence"[All Fields]) OR "residence s"[All Fields]) OR "residences"[All Fields]) OR "residency s"[All Fields]) OR "resident"[All Fields]) OR "resident s"[All Fields]) OR "residents"[All Fields]) OR "resides"[All Fields]) OR "residing"[All Fields]) AND (("interview"[Publication Type] OR "interviews as topic"[MeSH Terms]) OR "interviews"[All Fields])

**Translations**

**RESIDENCY:** "internship and residency"[MeSH Terms] OR ("internship"[All Fields] AND "residency"[All Fields]) OR "internship and residency"[All Fields] OR "residencies"[All Fields] OR "residency"[All Fields] OR "reside"[All Fields] OR "resided"[All Fields] OR "residence"[All Fields] OR "residence's"[All Fields] OR "residences"[All Fields] OR "residency's"[All Fields] OR "resident"[All Fields] OR "resident's"[All Fields] OR "residents"[All Fields] OR "resides"[All Fields] OR "residing"[All Fields]

**INTERVIEWS:** "interview"[Publication Type] .or. "interviews as topic"[MeSH Terms] .or. "interviews"[All Fields]

**ONLINE FELLOWSHIP INTERVIEWS**

Search: **ONLINE FELLOWSHIP INTERVIEWS** Sort by: **Most Recent**

"ONLINE"[All Fields] AND ((((("fellowship s"[All Fields] OR "fellowships and scholarships"[MeSH Terms]) OR ("fellowships"[All Fields] AND "scholarships"[All Fields])) OR "fellowships and scholarships"[All Fields]) OR "fellowship"[All Fields]) OR "fellowships"[All Fields]) AND (("interview"[Publication Type] OR "interviews as topic"[MeSH Terms]) OR "interviews"[All Fields])

**Translations**

**FELLOWSHIP:** "fellowship's"[All Fields] OR "fellowships and scholarships"[MeSH Terms] OR ("fellowships"[All Fields] AND "scholarships"[All Fields]) OR "fellowships and scholarships"[All Fields] OR "fellowship"[All Fields] OR "fellowships"[All Fields]

**INTERVIEWS:** "interview"[Publication Type] .or. "interviews as topic"[MeSH Terms] .or. "interviews"[All Fields]

**VIRTUAL FELLOWSHIP INTERVIEWS**

Search: **VIRTUAL FELLOWSHIP INTERVIEWS** Sort by: **Most Recent**

((((("virtual"[All Fields] OR "virtuality"[All Fields]) OR "virtualization"[All Fields]) OR "virtualized"[All Fields]) OR "virtualizing"[All Fields]) OR "virtuals"[All Fields]) AND ((((("fellowship s"[All Fields] OR "fellowships and scholarships"[MeSH Terms]) OR ("fellowships"[All Fields] AND "scholarships"[All Fields])) OR "fellowships and scholarships"[All Fields]) OR "fellowship"[All Fields]) OR "fellowships"[All Fields]) AND (("interview"[Publication Type] OR "interviews as topic"[MeSH Terms]) OR "interviews"[All Fields])

**VIDEOCONFERENCING FELLOWSHIP INTERVIEWS**

Search: **VIDEOCONFERENCING FELLOWSHIP INTERVIEWS** Sort by: **Most Recent**

(("videoconferenced"[All Fields] OR "videoconferencing"[MeSH Terms]) OR "videoconferencing"[All Fields]) AND ((((("fellowship s"[All Fields] OR "fellowships and scholarships"[MeSH Terms]) OR ("fellowships"[All Fields] AND "scholarships"[All Fields])) OR "fellowships and scholarships"[All Fields]) OR "fellowship"[All Fields]) OR "fellowships"[All Fields]) AND (("interview"[Publication Type] OR "interviews as topic"[MeSH Terms]) OR "interviews"[All Fields])

**Translations**

**VIDEOCONFERENCING:** "videoconferenced"[All Fields] OR "videoconferencing"[MeSH Terms] OR "videoconferencing"[All Fields]

**FELLOWSHIP:** "fellowship's"[All Fields] OR "fellowships and scholarships"[MeSH Terms] OR ("fellowships"[All Fields] AND "scholarships"[All Fields]) OR "fellowships and scholarships"[All Fields] OR "fellowship"[All Fields] OR "fellowships"[All Fields]

**INTERVIEWS:** "interview"[Publication Type] .or. "interviews as topic"[MeSH Terms] .or. "interviews"[All Fields]
